# Growth of Co Nanomagnet Arrays with Enhanced Magnetic Anisotropy

**DOI:** 10.1002/advs.201600187

**Published:** 2016-07-05

**Authors:** Laura Fernández, Maxim Ilyn, Ana Magaña, Lucia Vitali, José Enrique Ortega, Frederik Schiller

**Affiliations:** ^1^ Donostia International Physics Center20018Donostia‐San SebastiánSpain; ^2^ Fachbereich Physik und Zentrum für MaterialwissenschaftenPhilipps‐Universität Marburg35032MarburgGermany; ^3^ Centro de Física de Materiales (CSIC‐UPV‐EHU) and Materials Physics Center (MPC)20018San SebastiánSpain; ^4^ Departamento de Física Aplicada IUniversidad del País Vasco UPV/EHU20018San SebastiánSpain; ^5^ IkerbasqueBasque Foundation for Science48013BilbaoSpain

**Keywords:** array of nanomagnets, enhanced magnetic anisotropy, nanotemplates, self‐assembly

## Abstract

A trigon structure formed by submonolayer gadolinium deposition onto Au(111) is revealed as a robust growth template for Co nanodot arrays. Scanning Tunneling Microscopy and X‐Ray Magnetic Circular Dichroism measurements evidence that the Co nanoislands behave as independent magnetic entities with an out‐of‐plane easy axis of anisotropy and enhanced magnetic anisotropy values, as compared to other self‐organized Co nanodot superlattices. The large strain induced by the lattice mismatch at the interface between Co and trigons is discussed as the main reason for the increased magnetic anisotropy of the nanoislands.

## 1 Introduction

The implementation of nanomagnets for spintronics, magnetic storage,^1^ or quantum computing^2^ devices requires a thorough exploration of materials and fabrication methods. Self‐assembly emerges as an efficient approach to form such magnetic nanostructures with controlled size and uniform distributions over large distances.^3^ Specific magnetic properties of the nanomagnets, e.g., blocking temperature, easy‐axis of magnetization, or anisotropy energy, have to be tailored to fulfil the specific demands of the desired application. For this purpose, self‐organized growth of ferromagnetic metals has been typically performed on surfaces that exhibit spontaneous reconstructions or at vicinal surfaces. Among the most widely studied materials are Co and Fe, which grow, e.g., as atomically thick wires on vicinal Pt(997),^4^, ^5^ or as nanoclusters on Au(111) or Au(788).^6^, ^7^, ^8^, ^9^, ^10^, ^11^ Another possibility to create templates is profiting the lattice mismatch between a substrate and a monolayer‐thick film of a different material that can give rise to the formation of Moiré patterns which in many occasions are found to work as chemically and structurally stable templates for the growth of nanostructures. This has been observed for graphene,^12^ BN based structures,^13^, ^14^ as well as in rare‐earth/Au or Ag monolayer‐thick alloys.^15^, ^16^, ^17^, ^18^, ^19^, ^20^ Certainly, a further possibility is the use of strain‐relief dislocation networks as modulated substrates, as shown, e.g., in the case of Ag and Cu monolayers on Pt(111).^21^

In this work, we investigate a Gd‐Au hexagonal trigon phase as a growth template for arrays of Co nanomagnets. Trigon structures, defined as periodic patterns of triangular units, have been observed on reconstructed *fcc*(111) close packed metal surfaces that are affected by large tensile stress, e.g., Au or Pt.^22^, ^23^, ^24^, ^25^ The Gd‐Au trigon phase arises during the early stage of Gd growth on Au(111) at high temperatures. Here, the few deposited Gd atoms are embedded in the surface and modify the Au(111) herringbone reconstruction. This leads to an alteration of the structural interplay between the topmost, dense Au atomic layer and the gold crystal underneath. As we show here, the resulting network exhibits exceptional properties as template for the growth of self‐assembled Co nanomagnets. Using X‐ray Magnetic Circular Dichroism (XMCD) we study the magnetic properties of Co nanodot arrays that show a remarkably large anisotropy with the technologically important out‐of‐plane easy axis of magnetization. The dots can be defined as non‐interacting nanomagnets with hysteresis loops that are well described by the Stoner–Wohlfarth (SW) model.

## 2 Results and Discussion

### 2.1 Gd‐Au(111) Trigon Phase

**Figure 1:**
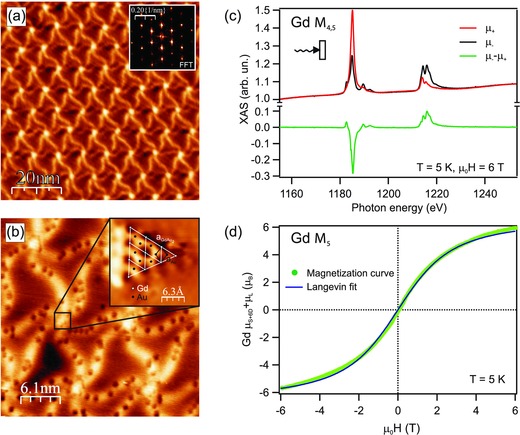
Scanning Tunneling Microscopy images showing a) the hexagonal Gd‐Au trigon network. Each bright node is formed by joining six dicommensuration lines (DL). b) Embedded Gd atoms appear as dark points in the trigon DL ($I_{t}$ = 0.3 nA, $U_{bias}$ = −1 V). The inset shows a zoom‐out of a trigon node, where the local GdAu_2_ atomic structure can be observed. Dark holes and bright protrusions, respectively, correspond to Gd and Au atoms ($I_{t}$ = 0.1nA, $U_{bias}$ = −1 V). c) X‐ray absorption measurements carried out with left and right circularly polarized light at μ_0_ *H* = 6 T. The difference signal is the XMCD signature of the measurement. d) XMCD magnetization curve taken at the maximum of the XMCD signal of the M_5_ line and the fit of the data with a single Langevin function.

Deposition of less than 0.1 monolayers (ML) of Gd at 690 K leads to the transformation of the Au(111) herringbone reconstruction into a periodic array of triangular structures (trigons), as shown in **Figure** **1**. The trigon nodes form a hexagonal network linked by wavy dicommensuration lines (DLs), with a lattice periodicity of (90 ± 6) Å.^16^ Gd atoms are incorporated at the trigon nodes and the DLs, where they can be individually imaged as dark holes, as shown in Figure 1b. The atomically resolved STM image of the trigon node in the inset defines a triangular structure that contains a honeycomb atomic arrangement of (5.1 ± 0.2) Å lattice constant. Additionally, the lattice is rotated by about 30° with respect to the trigon periodicity. The atomic structure in the node reveals, therefore, a $\sim \sqrt{3}\times \sqrt{3}$ R30° surface reconstruction with respect to the Au(111) surface, i.e., very similar to the superstructure observed in a GdAu_2_ monolayer.^16^, ^26^ Thus, trigon nodes can be considered the crystalline precursors that nucleate the continuous GdAu_2_ film at higher Gd coverage, having already a GdAu_2_ stoichiometry. In the trigon network shown in Figure 1 the number of Gd atoms present in each trigon superlattice unit cell is ≈30, where one third of them are located in the nodes, and the rest is embedded in the DLs. This results in a less than 5% atomic concentration of Gd at the surface, significantly smaller than the 33% concentration of the continuous GdAu_2_ layer.

The magnetic properties of the Gd‐Au trigon network are studied by XMCD at the Gd M_4,5_ absorption edge at 5 K. The magnetic field (μ_0_ *H*) is parallel to the propagation direction of the photons and is applied in out‐of‐plane (θ = 0°) or in‐plane (θ = 60°) geometry with respect to the (111) surface. Figure 1c shows the out‐of‐plane X‐ray absorption spectra at μ_0_ *H* = 6 T, using circular left and right polarized light. The lower curve (green line) is the difference of both spectra (XMCD spectrum). The magnetization curve in out‐of‐plane geometry in Figure 1d is obtained by recording the absorption intensity at the photon energy that corresponds to the maximum of the XMCD spectrum as a function of μ_0_ *H*. It displays a “S” shape without any signs of remanence. Furthermore, the same magnetization loop was obtained at in‐plane geometry, indicating a paramagnetic or unblocked superparamagnetic state. Magnetization curves were normalized to the total Gd moment μ_Gd_ at 6 T, being μ_Gd_ = μ_2*S* + 6*D*_ + μ_*L*_ the sum of the effective spin magnetic moment μ_2*S* + 6*D*_ and the orbital magnetic moment μ_*L*_ (see also Supporting Information). Both spin and orbital moments were obtained from the sum rules analysis of the absorption spectra,^27^, ^28^ which, in accordance with Hund's rule (*L* = 0 for Gd in its magnetic ground state), rendered μ_*L*_ = 0. In order to derive additional magnetic properties of the Gd atoms of the trigon phase, the magnetization curve measured at 5 K was fitted with a Langevin function^6^

(1) $$\mu_{\mathrm{Gd}}(B)=\mu \left[\coth\left(\frac{\mu NB}{k_{B}T}\right)-{\left(\frac{\mu NB}{k_{B}T}\right)}^{-1}\right]$$

where *B* = μ_0_ *H*. This fit yields the saturation magnetic moment μ = 6.95 μ_*B*_ and the average number of Gd atoms in each separate magnetic entity *N* = 1. The latter value means that Gd atoms do not form superparamagnetic or ferromagnetic clusters, where typically large *N* values are obtained.^6^, ^10^ In order to clarify if Gd atoms within the GdAu_2_ trigon nodes (*N*_node_ = 10 atom/cluster) have a different magnetic contribution than the isolated Gd atoms inside the DLs (*N*_isol_ = 1 atom/cluster), we have also tried the sum of two different Langevin functions. However, a fit with two functions (not shown) deviates strongly from the experimental results. Therefore, we conclude that all Gd atoms in the trigon phase, embedded in DLs or within triangular nodes, act as single paramagnetic impurities, in contrast with the ferromagnetic order found in the GdAu_2_ monolayer.^18^, ^19^ In the present case, the absence of ferromagnetism in the crystalline trigon nodes is likely due to their reduced lateral size.^29^

### 2.2 Co Dots on the Gd/Au(111) Trigon Phase

**Figure 2:**
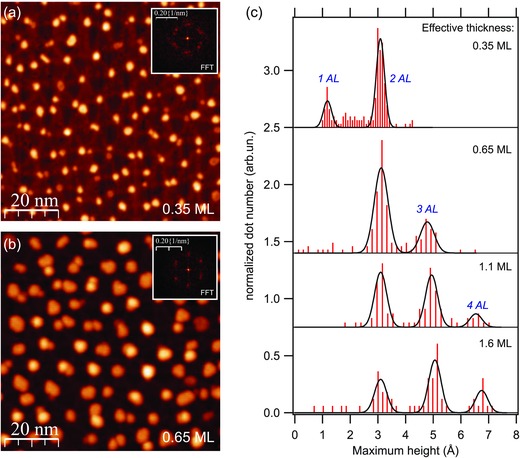
STM images showing Co nanodot arrays grown on a Gd‐Au trigon network for a) 0.35 ML and b) 0.65 ML coverage of Co. c) Statistical distribution of the height of Co nanodots. The maximum height distribution of the dots for different coverage appears indicated. The distribution was fitted by Gaussian functions that reflect the presence of different atomic layers in the dot.

Room‐temperature evaporation of Co on the trigon network leads to homogeneous and regular Co nanodot arrays with the same hexagonal symmetry as the template.^16^ At low Co coverage one may randomly observe two independent Co dots that grow at different edges of the same GdAu_2_ trigon node. The latter gives place to some disordered appearance, although all trigon lattice nodes are occupied, as seen for 0.35 ML in **Figure** **2**a. At this coverage the nanodot size distribution is broad, containing one and two atomic‐layer‐high (AL‐high) dots that display a pronounced volume difference. For higher coverage the nanodot volume and size distributions get more homogeneous with small variations (2–3 AL) of nanodot sizes, as shown for 0.65 ML coverage in Figure 2b. The insets of Figures 2a,b show the corresponding Fourier transformation of each STM image. They reveal an improvement in the periodicity of the nanodot array, i.e., ordering, at 0.65 ML, caused by the nanodot coalescence at the same trigon node that gives rise to more uniform dots. At much higher coverage (1.6 ML), coalescence of Co nanodots grown at neighboring trigon nodes takes place. In this situation the coalescence only affects few dots, but does not lead to large Co patches. An analysis of the nanodot height distribution between 0.35 and 1.6 ML is shown in Figure 2c. It is seen that the gradual increase of Co coverage leads to the formation of higher nanodots, which at 1.6 ML exhibit a maximum height of four AL. From the detailed analysis of the maximum height distributions one can detect that the interlayer distance within the nanodot amounts to (1.8 ± 0.2) Å, a value slightly smaller than the 2.0 Å found in Co/Au(111) surfaces.^30^ Such a decrease is expected for the growth of strained hexagonal Co perpendicular to the surface. Due to the increased lattice mismatch with respect to the Gd‐Au substrate, the Co in‐plane lattice is expanded and in order to maintain the Co unit cell volume, the interlayer distance has to be reduced. In the Supporting Information, further details about the Co dot structure are given. Moreover, an additional difference to the Co/Au system is the presence of 1 AL high nanodots on trigon networks. Monolayer‐thick nanodots were not observed on Co on Au(111),^6^, ^30^ although they form on GdAu_2_.^18^

**Figure 3:**
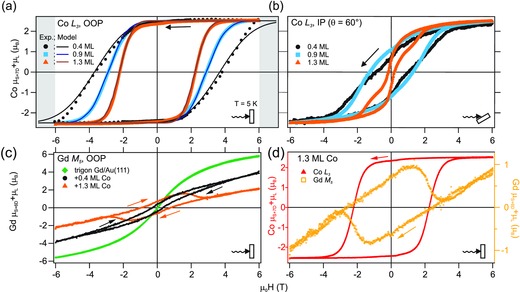
XMCD magnetization curves for several Co dot arrays on the trigon Gd/Au(111) system at the Co *L*_3_ absorption line for a) out‐of‐plane θ = 0° and b) in‐plane θ = 60° geometries. Markers are used for the experimental data while the continuous lines in (a) are the result of the rate equation model. c) Gd *M*_5_ magnetization curves in out‐of‐plane geometry as a function of the Co coverage. d) Comparison of the Co and the Gd signal of the Co‐covered regions for 1.3 ML thickness revealing the antiparallel coupling at the interface. The arrows indicate the direction of the change in the applied magnetic field in the two different branches of the magnetization loop. The first branch starts at μ_0_ *H* = +6 T and goes to +6 T, and the second branch goes back to +6 T.

Magnetic properties of the Co nanodot arrays were again studied by XMCD, which due to its chemical sensitivity offers the possibility to separately investigate the magnetic properties of Gd as well as Co atoms. The anisotropy of the Co nanodots was analyzed by both XMCD absorption spectra (sum rules analysis, see Supporting Information) and magnetization curves at different geometries. **Figure** **3**a shows the out‐of‐plane magnetization loops for various Co nanodot arrays measured at the maximum of the Co *L*_3_ XMCD signal. The loops have an almost square shape, specially well defined for 0.9 and 1.3 ML nanodot arrays, where the remanent magnetization is close to the magnetic saturation value. On the other hand, this square shape of the magnetization curve becomes less pronounced for low Co coverage (0.4 ML), due to the more heterogeneous dot size and volume distribution, as in fact observed by STM (see Figure 2). Moreover, it is seen that all out‐of‐plane hysteresis loops in Figure 3a display relatively high coercive fields (*H*_*c*_), e.g., *H*_*c*_ = 3.7 T for the 0.4 ML nanodot array, values that are substantially higher than the ones reported for both, continuous Co films grown on Au(111),^31^ and single‐domain Co islands grown on Au(788) (*H*_*c*_ = 0.5 T for 0.35 ML) at *T* = 10 K.^7^, ^10^

The out‐of‐plane easy axis of magnetization of the Co nanodots grown on trigons is confirmed by the hysteresis curves measured in IP geometry (θ = 60°), shown in Figure 3b. The magnetization loop measurements performed on 0.4 ML and 0.9 ML allow the description of these systems by the Stoner‐Wolfahrt (SW) model as non‐interacting nanomagnets.^32^ In particular the 0.9 ML array displays an excellent agreement, showing an in‐plane *H*_*c*_ value that is half the one measured in out‐of plane geometry. Furthermore, the remanent magnetization value in this geometry is found to be also half of the saturation magnetization, as predicted by the SW model. On the contrary, the SW model cannot explain the peculiar in‐plane hysteresis loop measured on the 1.3 ML array that displays a marked narrowing in the waist. The latter is thought to be connected with a drop of the first anisotropy constant due to the competition of shape and surface contributions, which counterbalance in Co/Au(111) between 4 and 5 layer thickness.^33^ Due to the influence of higher order anisotropy terms, in such films the magnetization reversal occurs in two steps, which may also occur for the nanodots in the present case,^34^ see the Supporting Information for more details.

In addition, Gd XMCD magnetization curves measured before and after Co evaporation reveal a gradual change from the paramagnetic behavior of the Gd atoms to a loop with hysteresis, as shown in Figure 3c. This transition is assigned to the direct Gd‐Co coupling at the interface.^35^, ^36^ In detail, the Gd magnetization curves after Co evaporation display two different contributions; a paramagnetic component with a “S”‐like shape that arises from uncovered Gd atoms, and a hysteresis curve from Gd coupled to Co atoms. The latter component increases with the size of the Co nanoparticle, as expected for a larger contact area between both materials. For a better visualization of the magnetic properties of the covered Gd atoms for the 1.3 ML sample, in Figure 3d the paramagnetic contribution of the uncovered Gd atoms was subtracted (around 20%, as deduced from the STM images). Then, the Gd magnetization curve results to be the mirror‐image of the Co hysteresis loop, plus a linear slope. Specially at μ_0_ *H* = 0 the remanent magnetic signal has the opposite sign for Co and Gd. These facts evidence an antiferromagnetic (AFM) coupling between Gd and Co atoms. A similar AFM coupling was already observed for Co nanodots grown on GdAu_2_ layers,^18^ although in the present case, however, the absence of magnetic saturation in the Gd hysteresis loops points to a very strong AFM coupling (see Figure 3d). Note the difference with the continuous GdAu_2_ substrate, where magnetic ordering in the Gd substrate lattice exists prior to Co evaporation.^18^ In the trigon network, in contrast, Gd atoms only become magnetically arranged by interacting with magnetic Co.

In order to quantify the magnetic anisotropy energy (MAE) of the Co nanodot array we numerically fit the XMCD out‐of‐plane magnetization curves using the rate equation model^10^, ^19^ (see also Supporting Information). The latter takes into account the thermal excitation of the magnetic moments, and therefore it gives a more realistic description of small magnetic nanodots. In this model the MAE distribution is supposed to be Gaussian, whose characteristic parameters (mean anisotropy energy *K* and full width at half maximum (FWHM) values) are obtained from the fitting of each Co nanodot array. The fitting procedure is simplified by assuming a fixed magnetic moment value for an *N*‐atom dot *M* = *N* · μ_*at*_.^10^ The magnetic moment per Co atom μ_*at*_ = μ_*L*_ + μ_*S*_ is extracted from the sum rule analysis, and *N*, the number of Co atoms per dot is estimated from the STM analysis, namely *N* = 190, 630, and 880 atoms for 0.4, 0.9, and 1.3 ML nanodot arrays, respectively (see the Supporting Information for determination of these values). The resulting hysteresis loops obtained from the model are included in Figure 3a as solid lines. They agree very well with the experimental magnetization loops (markers). The obtained *K* and FWHM values from the fitting process are summarized in **Table** **1**. For comparison, *K* and FWHM values of Co nanodots grown on different substrates are added. The total anisotropy for the Co nanoclusters has several contributions, the most important here are magneto‐crystalline (including surface/interface) and shape anisotropy *K*_sh_. This scenario differs from Co bulk, where the latter one is predominant. Co dots grown on the Gd‐Au trigon substrate reveal quasi‐hexagonal shapes with a form factor (height to diameter ratio) smaller than 0.1. Therefore their geometry can be approximated as oblate spheroids with a shape anisotropy that is similar to an infinite plane^37^ having a value of $K_{sh}=2\pi M^{2}$.^32^ By taking the magnetization of bulk hcp Co (1400 emu cm^−3^) one obtains $K_{sh}=-0.085$ meV atom^−1^. More recent investigations have shown that the shape anisotropy calculated as a sum of contributions of discrete magnetic dipoles differs from the value predicted by the continuum approximation, such that it varies with thickness rather than with a form factor.^38^ Nevertheless, this correction change the given value by less than 10%, and therefore the shape anisotropy is not responsible for the large magnetic anisotropy observed in this work and shown in Table 1.

**Table 1:** Magnetic anisotropy energy distributions for the trigon phases and similar Co dots on Au.^10^ The *K*_*p*_ values on Au(111) cannot be exactly determined due to the unknown dot morphology^39^

Substrate	Thickness	*N*	*K* [meV]	FWHM [%]	*K* [meV at.^−1^]	*K*_*p*_ [meV at.^−1^]
Gd trigon	0.4	190	84	64	0.44	1.22
Gd trigon	0.9	630	181	54	0.29	1.32
Gd trigon	1.3	880	192	42	0.22	1.08
Au(788)^10^	0.35	120	26	200	0.22	0.5
Au(788)^9^	0.75	240	75	35	0.31	0.8
Au(11,12,12)^3^	1.1	600	110	–	0.18	0.9
Au(111)^7^	–	1500	155	170	0.10	<0.8
Au(111)^7^, ^10^	1.5	4000	360	60	0.09	<0.8

It is important to note that nearly the complete MAE distribution of the Co dots on the trigon phase is positive, i.e., almost all nanodots reveal an out‐of‐plane anisotropy, thereby the use of the rate equation model is validated. The observed MAE distributions becomes sharper when the volume of the Co nanodot increases, which is assigned to a more homogeneous volume distribution for larger nanodots, as seen in the STM analysis. Notably, *K* is not observed to scale with the number of atoms *N* in the dot, but rather with $\sqrt{N}$, in a similar way as observed for Co nanodots on Au(788) or Pt(111).^9^, ^40^ This can be explained by an anisotropy constant *K* that is mainly determined by perimeter atoms, which have lower coordination. In such case, *K* can be written as *K* = *pK*_*p*_, with *p* being the number of perimeter atoms, and *K*_*p*_ the anisotropy value per perimeter atom. Considering the number of perimeter atoms deduced from the STM images (see Supporting Information), we obtain that *K*_*p*_ is approximately (1.25 ± 0.2) meV/atom for 0.4 ML and 0.9 ML nanodot arrays, and it is slightly reduced to *K*_*p*_ = (1.1 ± 0.2) meV/atom for the 1.3 ML array. Thus, *K* is notably larger in Co nanodots grown on trigons than in those grown on Pt(111), Au(788), or Au(11,12,12) [0.8–0.9 meV/atom].^3^, ^9^, ^40^

The magneto‐crystalline (MC) anisotropy of bulk hcp Co is 0.035 meV/atom,^32^ which is one order of magnitude lower than the values of the nanodots in Table 1. Larger MAE values that are observed in nanostructures with reduced size are mainly attributed to the MC contribution produced by reduced coordination of the surface/rim atoms which avoid the quenching of their orbital moments.^9^, ^41^ Furthermore the Au atoms surrounding the Co dots have to be taken into account. Encapsulation of Co dots by Au atoms have been found to rise the MAE,^42^ either due to a polarization of the neighboring Au atoms^43^ or due to the modified strain in the Co lattice.^42^ Also in epitaxial Ni films on Cu(100) a strong enhancement of the MC anisotropy caused by the interface strain was reported.^44^ For the Co dots on the trigons considered here, the shape and surrounding of the clusters is similar to other Co dots on flat and vicinal Au(111) surfaces mentioned in Table 1. Therefore the enhanced *K*_*p*_ values may arise from a stronger interface strain in the trigon template. This is supported by the fact that in pseudomorphically grown Co nanodots, a large lattice strain would be present at Co atoms, specially on top of trigon nodes. The hcp Co in‐plane lattice constant of 2.51 Å is small compared to the 2.88 Å Au surface atom distance in Au(111), but it is even smaller compared to the 3.00 Å in‐plane nearest‐neighbor distance in the GdAu_2_ trigon nodes. Also at areas outside the nodes, where single Gd atoms are incorporated into the DLs, a larger nearest neighbor distance compared to pure Au(111) occurs. Finally, *K* is observed to become smaller for large, i.e., thick nanodots, as expected for an increasing proportion of perimeter atoms at the second and third nanodot layers, which are less influenced by interface strain.

## 3 Conclusions

The Gd‐Au(111) trigon phase prepared by evaporation of Gd on an Au(111) surface at 690 K is revealed as an innovative template for the growth of ferromagnetic nanodot arrays. Co nanodots grown on this surface are found to behave as independent nanomagnets with a clear out‐of‐plane easy axis of magnetization, as it is unambiguously shown by the hysteresis loops measured using XMCD. Using the rate equation model, especially designed for the study of magnetic nanodot systems, it is concluded that the nanodots grown here exhibit relatively narrow magnetic anisotropy energy distributions and enhanced anisotropy values, as compared to similar nanodots grown on more conventional surfaces such as Au(788) or Pt(111). A detailed STM and XMCD analysis of the trigon structure reveals a network of crystalline GdAu_2_ nodes that due to their reduced size behave paramagnetically, without any sign of superparamagnetism. However, the large lattice mismatch at the Co/GdAu_2_ interface is thought to induce a high interface strain that enhances the magnetic anisotropy of the Co nanodots grown on top. All in all, the trigon phase of the Gd‐Au(111) system appears as an innovative and promising template to self‐organize different magnetic nanostructures with exotic and improved anisotropy properties. Moreover, the use of new metals in order to produce different trigon phases with different structural properties is suggested as a new objective for the design of new templates.

## 4 Experimental Section

The morphology of the Gd‐Au trigon network and Co islands grown on top was studied at room temperature by STM in San Sebastian and at the SOLEIL synchrotron. The samples were prepared in situ under ultrahigh vacuum conditions (*p* < 2 × 10^−10^ mbar). Growth of Gd on Au(111) was carried out by exposing the Au(111) substrate, held at 690 K, to a pure Gd vapor for a short time. The subsequent growth of Co was carried out at 300 K, with a Co coverage that was varied between 0.2 and 1.6 ML (STM) and 0.4 and 1.3 ML (synchrotron measurements), respectively. The coverage in this case is given with respect to the same amount of Co deposited onto a clean Au(111) crystal and accounting for the initial double layer growth of Co/Au(111),^30^ see the Supporting Information section for a detailed description of the STM analysis.

X‐ray magnetic circular dichroism (XMCD) measurements were carried out at the DEIMOS beamline of the French SOLEIL synchrotron facility using a 98%–99% circularly polarized light from a helical ondulator.^45^ The measurements were undertaken between 3.5 and 5 K with a variable magnetic field up to ±6 T pointing along the direction of the synchrotron light. Experiments were carried out for normal (θ = 0°, out‐of‐plane) and grazing incidence (θ = 60°, in‐plane geometry). Absorption spectra were acquired at the Co *L*_2,3_ and Gd *M*_4,5_ edges (total electron yield). Element sensitive magnetization loops were measured recording the maximum of the XMCD asymmetry signal at the Co *L*_3_ and Gd *M*_5_ absorption edges as a function of the magnetic field. Sum rules were used to obtain orbital μ_*L*_ and effective spin μ_2*S* + *nT*_ moments of Co (*n* = 7) and Gd (*n* = 6) from XMCD spectra taken at μ_0_ *H* = 6 T. In the latter case the effective spin moment of the rare earths was defined as $\mu_{S_{\mathrm{eff}}}$ = 2*S*_*z*_ + 6*T*_*z*_, following Thole's criteria.^46^ For Gd, the number of holes in the *f*‐shell was considered as 7, whereas for Co, the number of holes in the *d*‐shell was set to 2.49.^10^ The sum μ_*L*_ + μ_2*S* + *nT*_ was used to normalize the XMCD magnetization curves.

## Supporting information

As a service to our authors and readers, this journal provides supporting information supplied by the authors. Such materials are peer reviewed and may be re‐organized for online delivery, but are not copy‐edited or typeset. Technical support issues arising from supporting information (other than missing files) should be addressed to the authors.

Supplementary
