# Supplementary material for: Growth of Co Nanomagnet Arrays with Enhanced Magnetic Anisotropy
Source: Adv Sci (Weinh). 2016 Jul 5;3(9):1600187. doi: 10.1002/advs.201600187 (PMC5039974; doi:10.1002/advs.201600187)
Supplement: Supplementary file 1 — Supplementary [file ADVS-3-0h-s001.pdf]

Copyright WILEY-VCH Verlag GmbH & Co. KGaA, 69469 Weinheim, Germany, 2016.

ADVANCED SCIENCE
Open Access

Supporting Information

for *Adv. Sci.,* DOI: 10.1002/advs.201600187

Growth of Co Nanomagnet Arrays with Enhanced Magnetic Anisotropy

*Laura Fernández, Maxim Ilyn, Ana Magaña, Lucia Vitali, José Enrique Ortega, and Frederik Schiller**

DOI: 10.1002 /advs.201600187
Article type: Full paper

Supporting Information

Growth of Co nanomagnet arrays with enhanced magnetic anisotropy

*Laura Fernández, Maxim Ilyn, Ana Magaña, Lucia Vitali, José Enrique Ortega, and Frederik Schiller**

Dr. L. Fernández, Prof. J.E. Ortega
Donostia International Physics Center, 20018 Donostia/San Sebastián, Spain

Dr. L.Fernández, Dr. F. Schiller.
Philipps Universität Marburg, Fachbereich Physik, 35032 Marburg, Germany

Dr. M. Ilyn, Dr. A.Magaña, Dr. L.Vitali, Prof. J.E. Ortega, Dr. F. Schiller
Centro de Física de Materiales, 20018 Donostia/San Sebastián, Spain

Dr. A.Magaña, Prof. J.E. Ortega
Univeridad del País Vasco, Dep. Fisica Aplicada I, 20018 Donostia/San Sebastián, Spain

Dr. L.Vitali
Ikerbasque, Basque Foundation for Science, 48013, Bilbao, Spain

E-mail: frederikmichael.schiller@ehu.es

Keywords: nanotemplate, self-assembly, array of nanomagnets, enhanced magnetic anisotropy

1 Structure of Co nanodots

Scanning Tunneling Microscopy measurements carried out on Co nanodots grown on a Gd-Au-trigon surface reveal a characteristic hexagonal shape with three-fold symmetry, with long and short segments opposite to each other. Opposite segments are thought to correspond to step-edges with different crystal orientation. This leads to different step-flow-growth rates upon Co deposition, and hence to irregular hexagonal dot shapes. The quasi-hexagonal shape is already detected at lower Co coverage, as shown in **Figure S**Error! Reference source not found., which displays the nanodot array after evaporation of 0.35 ML of Co. The inset shows a closer view of one of the dots, revealing the hexagonal morphology commented above. Additionally, the closed-packed directions $\langle 1\bar{1}0 \rangle$ of the Au(111) surface are indicated, concluding that the dots mainly grow following a well-defined orientation of the substrate. A similar (distorted) hexagonal shape has been already shown for Co nanodots grown on Au(111) surfaces [**Error! Reference source not found.**]. In the trigon case the average

substrate lattice parameter is larger compared to that of the pure Au(111) surface. This likely leads to a larger strain in the Co nanodot, but does not affect its hexagonal atomic arrangement.

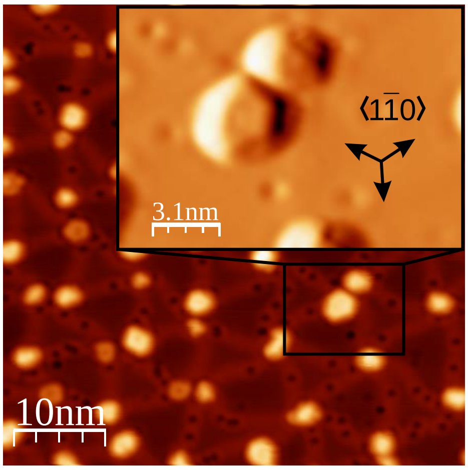

Figure S1. STM image of the 0.35 ML Co nanodot array on the trigon Gd-Au substrate. The inset is a derivative image of the marked area where the hexagonal structure of the Co dot can be observed similar as in Co/Au(111).[1]

2 Sum rules analysis of the Co dots

The orbital L_z (μ_L) and effective spin $S_{eff} = S_z + 7T_z$ ($\mu_{S_{eff}}$) magnetic moments projected onto the photon incidence direction z is obtained by applying the XMCD sum rules[**Error! Reference source not found.**, **Error! Reference source not found.**]. In this case S_{eff} includes the contribution from the spin dipole moment T_z. The number of holes in the *d*-band in the case of Co is set to the corresponding bulk value of 2.49 [**Error! Reference source not found.**].

A critical point for the correct estimation of the Co L$_{2,3}$ XMCD signal and the consequent application of the sum rules is the strong background produced by the contribution of the extended X-ray absorption fine structure (EXAFS) variations of the Au substrate. The correct value has been achieved subtracting this background measured on the clean Au surface. In **Figure S**Error! Reference source not found. we show the obtained μ_L values as a function of the angle of the magnetic field with respect to the surface normal and coverage of Co nanodots. These XMCD measurements were carried out at T = 5 K and $\mu_0 H$ = 6 T of applied magnetic field. The extracted $\mu_L(\theta)$ values can be decomposed as

$$\mu_L(\theta) = \mu_L^{\perp} + (\mu_L^{\parallel} - \mu_L^{\perp})\sin^2\theta \qquad (1)$$

where $\mu_L^{\parallel}$ and $\mu_L^{\perp}$ denote the parallel and perpendicular component of μ_L. The difference $\mu_L^{\perp}$ - $\mu_L^{\parallel}$ renders the magnetocrystalline anisotropy energy per atom [**Error! Reference source not found.**, 6, 7, 8]. Independently of the Co nanodot size, we find a constant value of $\mu_L^{\perp}$ - $\mu_L^{\parallel}$ =(0.07±0.01) μ_B per Co atom, which is significantly smaller than the value found for Co dots self-organized on Au crystal surfaces (0.11 μ_B [**Error! Reference source not found.**, **Error! Reference source not found.**, 7]). The positive value of μ_L reflects the out-of-plane anisotropy of the Co nanodots grown on the trigon template. The magnetocrystalline anisotropy per atom k is linked to the μ_L anisotropy by [6, 7, **Error! Reference source not found.**, **Error! Reference source not found.**]

$$k = \alpha \frac{\xi}{4}(\mu_L^{\parallel} - \mu_L^{\perp}) \qquad (2)$$

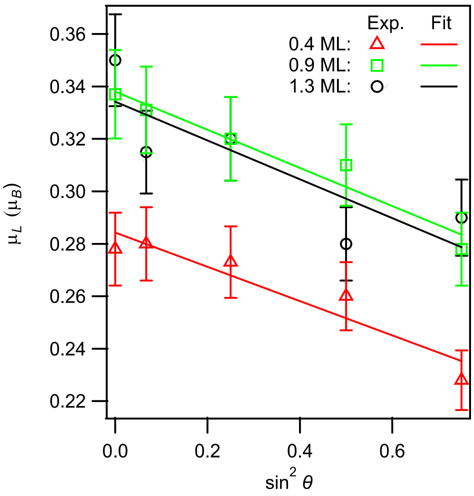

Figure S2. Magnetic moment μ_L as a function of the angle θ between the magnetic field and the surface normal. All μ_L values were derived from sum rule analysis.

with the Co spin-orbit coupling constant ξ=70 meV [**Error! Reference source not found.**]. α accounts for the fact that XMCD usually overestimates the magnetocrystalline anisotropy and is found here to vary between α = 0.14 for 0.4 ML to α = 0.23 for 1.3 ML Co coverage confirming the overestimation of the anisotropy by the XMCD spectra. Following the same formalism described above the spin moment $\mu_{S_{eff}}$ can be deduced as $\mu_{S_{eff}}$ = (2.19±0.15) μ_B per Co atom, value that is considerably larger than the 1.7 μ_B value found for Co dots on vicinal Au(111) [**Error! Reference source not found.**, **Error! Reference source not found.**].

3 Calculation of the total number of atoms and perimeter atoms of the nanodots

The analysis of the magnetic moment per atoms strongly relies on the correct estimation of the Co dot coverage, of their height and size. For this reason during the data analysis, considerable attention has been paid in comparing the coverage of Co nanodots achieved in the two laboratory located in San Sebastian and at the synchrotron Soleil. More specifically the dot distributions have been analyzed from the STM images measured in our laboratory in San Sebastian. The dot thickness and size distribution of the nanodot arrays measured by XMCD in SOLEIL were also analyzed by STM and compared with the one taken in our home laboratory. From these investigations that we have carried out, the total coverage and the number of atoms/dot have been estimated. All images were evaluated with the WSXM software [10]. First, STM images showing single terraced were flattened, then the **Flooding** tool was used for further analysis. This tool provides information like the number of islands, the occupied area, perimeter, volume etc. as a function of the flooding level. For calculating the effective thickness, the nanodot array is flooded with a minimum flooding level that must cover all the substrate, including the dicommensuration lines. This leads to the relative area of the sample that remains uncovered by that flooding level, and that corresponds to the area of the first atomic layer (AL) of the nanodots. In order to consider the contribution to the effective thickness of nanodots with 2 and more ALs, the flooding level is gradually increased by the Co bulk interlayer distance, obtaining with each new flooding level the area occupied by the different ALs of the nanodots. The sum of all the relative areas obtained for the different ALs included in the nanodots (typically from 1 - 4 ALs) provides the total coverage.

The total number of atoms per dot N is obtained from the nanodot volume distribution, which is extracted from the STM images. As an example, the analysis of 0.65 ML nanodot array is shown in **Figure S**Error! Reference source not found.. A gaussian fit to the volume distribution results in a mean dot volume

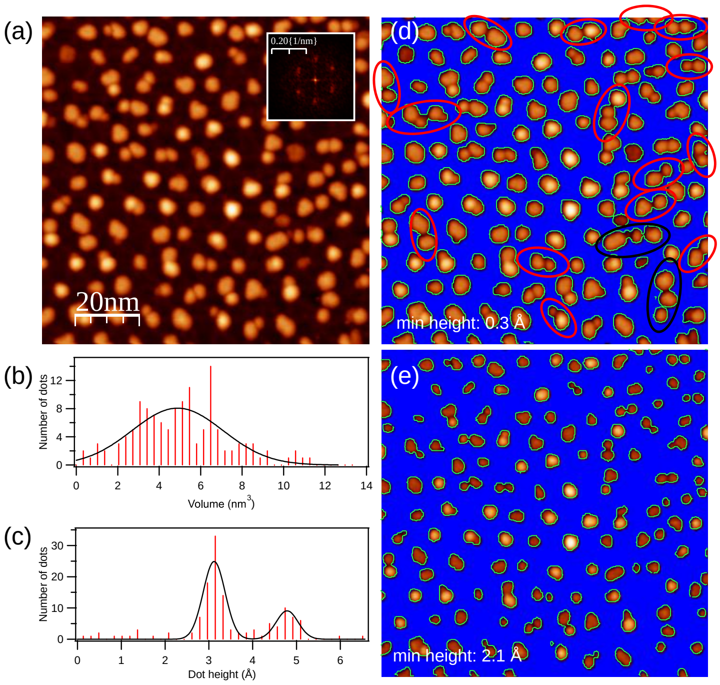

Figure S3. Example of the analysis of the dot distribution from the STM measurements for a coverage of 0.65 ML. (a): STM image of the dot distribution, the inset shows the Fourier transform. (b),(c): Volume and Maximum dot height histograms with corresponding single and double peak gaussian fits, respectively. (d),(e): Analysis of the STMimage with the “Flooding” tool of the indicated height levels, revealing separated but not resolved dots due to the STM tip broadening that can be separated at a different height level.

of 4.9 nm^3 with a full width at half maximum (FWHM) of 5.2 nm^3. This volume estimation is affected by two contributions: (i) neighboring dots can appear apparently joined due to tip broadening, and (ii) dots located at the edge of the STM measurement are not completely imaged. In order to observe the first effect, in Figure S**Error! Reference source not found.**(d) we mark the joined dots (due to the tip broadening) by red and black ellipses. The average dot volume is nearly unaffected since both effects occur at opposite sides of the distribution and in a similar number, only the FWHM is artificially widened. By using the Co density we obtain the total number of atoms/dot *N*, in this example 450 atoms. All the other nanodot distributions were analyzed in the same way. Figure S**Error! Reference source not found.**(a) shows the coverage of the nanodot arrays grown in our home laboratory and the ones taken at SOLEIL. Both data sets follow the expected linear increase with evaporation time. In Figure S**Error! Reference source not found.**(b) we plot the number of atoms/dot as a function of the coverage (filled red circles). Three different regions are observed: (i) at low coverage the small dots grow at the same trigon node; (ii) at intermediate coverage the small dots located at the same trigon node are coalesced and therefore a strong increase in the mean dot volume and hence in the number of atoms per dot; (iii) at higher coverage dots from different nodes start to coalesce forming larger islands. The number of atoms/dot in the nanodot arrays prepared in SOLEIL is obtained by linear interpolation of the data in Figure S**Error! Reference source not found.**(b). These values are shown in Table 1 of the main text. For the 0.4 ML thickness, 10% of the dots have one AL height, 80% two ALs, and 10% three AL. In the case of 0.9 ML thickness 60% of the dots are 2 AL high and 40% have 3 ALs. For a thickness of 1.3 ML, the dot distribution is approximated by 30% double ALs, 50% have 3 ALs, and 20% quadruple ALs height.

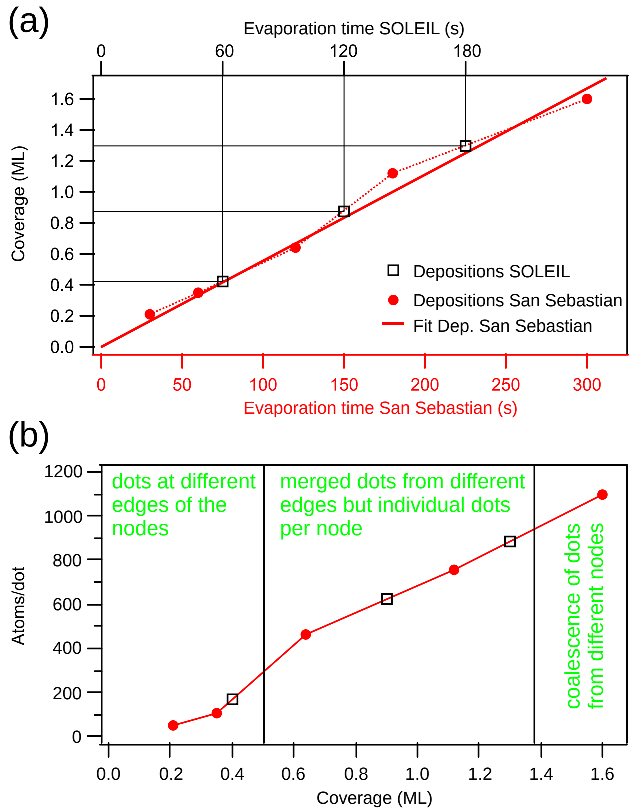

Figure S4. Analysis of the dot distribution fromthe STM measurements in San Sebastian and at SOLEIL. (a): Coverage of the nanodots as a function of evaporation time. (b): Number of Co atoms/dot as a function of coverage.

For these dots distributions the number of low coordinated atoms of the dots, hereafter perimeter atoms, can be estimated taking into account the height (*m* monolayer) and area distributions of the analyzed Co dots. Similarly to previous works [**Error! Reference source not found.**, **Error! Reference source not found.**] we assume the islands as cylindrically shaped. The number of atoms p_s at the cylinder barrel for a cylindrically shaped island of *m* layers and number of atoms *N* is $p_s = 2\sqrt{m\pi N}$. From the dot size distribution we use the relative percentage of the height distribution per layer a_m. Then the total number of perimeter atoms is

$$p = \sum_{m=1}^{m_{max}} a_m 2\sqrt{m\pi N}. \qquad (3)$$

From the dot volume and area distributions we calculate for the nanodot arrays prepared in SOLEIL the number of perimeter atoms as $p_{0.4ML}$ =69, $p_{0.9ML}$ =137, and $p_{1.3ML}$ =178, respectively. The relative error of the atoms/dot value is estimated to be 30%, and the error for the perimeter atom number is ≈15%, since it scales with the square root of the dot atomic number.

4 Magnetization loops of 1.3 ML dots

The in-plane hysteresis loop measured on the 1.3 ML Co nanodot array reveals a peculiar narrowing in the waist that can be explained considering the theoretical model developed by Millev *et. al.* [**Error! Reference source not found.**]. The latter predicts that a decrease of the first anisotropy constant due to the competition between the shape and the interface contributions to the anisotropy leads to a stronger influence of higher order anisotropy terms and provoke a two-step magnetization reversal. In general, an uniaxial magnetic particle has a magnetic anisotropy energy (MAE) that can be expanded considering two anisotropy terms

$$F_A = K_1 \sin^2 \varphi + K_2 \sin^4 \varphi$$

φ denotes the angle between the magnetic moment and the easy axis of the system, here the out-of-plane direction and K_1 and K_2 are the anisotropy constants [**Error! Reference source not found.**]. The systems presents an out-of-plane easy axis if $K_1 > 0$, $K_1 > -2K_2$, and if additionally $K_2 < 0$, $-6K_2 > K_1 > -2K_2$, then the magnetization reversal in the field perpendicular to the easy axis takes place in two steps. At low fields, the magnetic moment rotates gradually off the easy-axis direction, but at a certain critical value of the field, it flips into the field direction. This later process is a first order phase transition which is accompanied by a magnetic field hysteresis. The predicted magnetization loop is shown schematically in Figure 6 of Ref. [**Error! Reference source not found.**]) and is very similar to the in-plane magnetization curve of the 1.3 ML Co nanodot system on the trigons investigated here. Experimental values of the anisotropy constants of Co/Au(111) ultrathin films are also reported by the same authors [**Error! Reference source not found.**]. It was found that for a coverage lower than 5 ML, $K_1 > 0$ and $K_2 < 0$, and if the thickness is close to 2 ML $K_1 \approx -3K_2$. These values match the requirements for the first order magnetization reversal process in the in-plane field but, to our knowledge, these measurements have never been reported for the Co islands grown on Au(111) single crystal. It turns out that in our case only the sample with 1.3 ML effective thickness of Co meets the requirements and demonstrates the first order magnetization reversal when magnetized at 60° off the normal to the surface plane. If (as we claim here) the anisotropy comes mainly from perimeter atoms, then the effective K_1 (per total number of atoms) scales as 1/*R*, being *R* the dot radius. In the cases of the smaller islands (0.4 and 0.9 ML Co samples) K_1 is already too high and we don't observe the contribution of K_2.

5 Rate equation model

The rate equation model (REM) is used to describe the magnetization reversal of magnetic nanoparticles with an uniaxial magnetic anisotropy [**Error! Reference source not found.**]. In contrast to the Stoner-Wohlfarth (SW) theory this model does not allow the calculation of the equilibrium orientation of the magnetic moment. Instead REM considers magnetic particles as a system which has only two equilibrium orientations of the magnetic moment and whose energy is approximated by the two potential wells separated by a barrier. The magnetization

reversal in the REM is a random event with a probability depending on the temperature and the applied magnetic field. The advantage of the REM is an implicit dependence of the coercive field on the temperature which lacks the SW theory. Moreover, the rate equation model is an one- dimensional model that cannot be used to calculate the rotation of the magnetic moment in the nanoparticles when the magnetic field is applied perpendicular to the easy-axis. We applied the REM to all experimental magnetization loops in order to obtain the parameters of the anisotropy distribution for each sample in the same way as it was done in the work [**Error! Reference source not found.**]. The anisotropy distributions obtained from the fitting of the experimental magnetization curves are shown in **Figure S**Error! Reference source not found.. The resulting fit of the magnetization curve is included in Figure 3, the values can be found in Table 1, both in the main text.

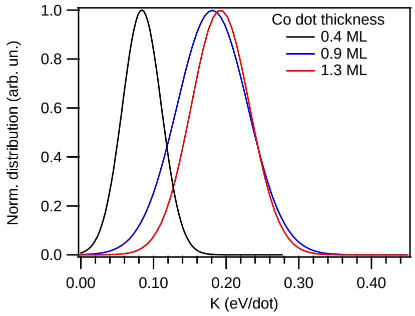

Figure S5. Anisotropy distributions that fit the experimental out-of-plane magnetization curves via the rate equation model.

References

[1] B. Voigtländer, G. Meyer, N.M. Amer, *Phys. Rev. B*, **1991**, *44*, 10354.
[2] P. Carra, B.T. Thole, M. Altarelli, X. Wang, *Phys. Rev. Lett.*, **1993** *70*, 694.
[3] C.T. Chen, U. Udzerda, H.J. Lin, N.V. Smith, G. Meigs, E. Chaban, G.H. Ho, E. Pellegrin, F. Sette, *Phys. Rev. Lett.*, **1995**, *75*, 152.
[4] S. Rohart, V. Repain, A. Tejeda, P. Ohresser, F. Scheurer, P. Bencok, J. Ferré, S. Rousset, *Phys. Rev. B*, **2006**, *73*, 165412.
[5] T. Koide, H. Miyauchi, J. Okamoto, T. Shidara, A. Fujimori, H. Fukutani, K. Amemiya, H. Takeshita, S. Yuasa, T. Katayama, Y. Suzuki, *Phys. Rev. Lett.*, **2006**, *87*, 257201.
[6] P. Bruno, *Phys. Rev. B*, **1989**, *39*, 865.
[7] D. Weller, J. Stöhr, R. Nakajima, A. Carl, M.G. Samant, C. Chappert, P. Mégy, P. Beauvillain, P. Veillet, G.A. Held, *Phys. Rev. Lett.*, **1995**, *75*, 3752.
[8] J. Stöhr, *Journal of Magnetism and Magnetic Materials*, **1999**, *200*, 470.
[9] N. Weiss, T. Cren, M. Epple, S. Rusponi, G. Baudot, S. Rohart, A. Tejeda, V. Repain, S. Rousset, P. Ohresser, F. Scheurer, P. Bencok, H. Brune, *Phys. Rev. Lett.*, **2005**, *95*, 157204.
[10] I. Horcas, R. Fernández, J.M. Gómez-Rodríguez, J. Colchero, J. Gómez-Herrero, A.M. Baro, *Review of Scientific Instruments*, **2007**, *78*, 013705.
[11] Y.T. Millev, H.P. Oepen, J. Kirschner, *Phys. Rev. B*, **1998**, *57*, 5848.
[12] A. H. Morrish *The Physical Principles of Magnetism*, Wiley-IEEE Press New York **2001**.
